# CRISPR-Cas9 Mediated Genome Editing in *Bicyclus anynana* Butterflies

**DOI:** 10.3390/mps1020016

**Published:** 2018-05-14

**Authors:** Tirtha Das Banerjee, Antónia Monteiro

**Affiliations:** 1 Department of Biological Sciences, National University of Singapore, 14 Science Drive 4, Singapore 117543, Singapore; 2 Yale-NUS College, 10 College Avenue West, Singapore 138609, Singapore

**Keywords:** CRISPR-Cas9, sgRNA, *Bicyclus anynana*

## Abstract

CRISPR-Cas9 is revolutionizing the field of genome editing in non-model organisms. The robustness, ease of use, replicability and affordability of the technology has resulted in its widespread adoption among researchers. The African butterfly *Bicyclus anynana* is an emerging model lepidopteran species in the field of evo-devo, with a sequenced genome and amenable to germ line transformation. However, efficient genome editing tools to accelerate the pace of functional genetic research in this species have only recently become available with CRISPR-Cas9 technology. Here, we provide a detailed explanation of the CRISPR-Cas9 protocol we follow in the lab. The technique has been successfully implemented to knock-out genes associated with eyespot development and melanin pigmentation.

## 1. Introduction

In the past three decades, discovery and engineering of tools such as zinc finger nucleases (ZFNs), transcription-activator like effective nucleases (TALENs) and clustered regularly interspaced short palindromic repeats (CRISPR) have revolutionized the field of genome engineering [1,2].

Zinc finger nucleases were the first-generation tools for genome engineering. The ability of zinc finger domains to recognize specific DNA sequences was first described in *Xenopus laevis* [3]. In 1996, Kim et al. reported the development of a fusion protein where they managed to link zinc finger domains to the cleavage domain of a FokI endonuclease. The resulting dimers showed the ability to cleave DNA at specific sites [4]. Zinc finger nucleases have been utilized to edit genomes of a wide array of model organisms including fruit flies [5,6], silk moths [7], zebrafish [8,9], *Arabidopsis* [10], rats [11] and pigs [12] and non-model organisms such as the cricket *Gryllus bimaculatus* [13] and the butterfly *Danaus plexippus* [14]. Although ZFNs showed great promise, the difficulty in designing and testing ZFNs and the associated costs restricted the use of this technology to only a few labs [1,15,16].

The next wave of genome engineering involved the hybrid proteins TALENS. Scientists studying phytopathogenic bacteria *Xanthomonas* in 2009 discovered transcription activator-like effectors (TALEs) proteins, capable of recognizing specific sites of DNA [17]. The following year, another group of researchers developed a new class of hybrid proteins called TALENs, which contains the site-specific TALEs fused to the catalytic site of the FokI endonuclease [18]. TALENs are much easier to synthesize, have higher sequence specificity compared to ZFNs, and do not require tedious validations [15,19]. This second-generation tool led to rapid acceleration in genome engineering and was used to study model organisms such as fruit flies [20], silk moths [21,22] nematodes [23], zebrafish [24], pigs [25] and frogs [26], as well as non-model organisms such as mosquitos *Anopheles gambiae* [19] and *Aedes aegypti* [27]; the moth *Ostrinia furnacalis* [28]; and the cricket *Gryllus bimaculatus* [13]. As with ZFNs, a significant limitation to TALENs include the technical challenge and cost associated with the development of custom designed proteins [16].

The year 2012 marked a major leap in the field of genome engineering when the labs of Jennifer Doudna and Emmanuelle Charpentier jointly published their paper describing an RNA-programmable nuclease that can cut DNA at targeted sites [29]. This nuclease (Cas) is encoded next to bits of DNA taken from previous invading viruses, or spacer sequences, whose RNA sequences guide Cas to target complementary DNA sequences of invading viruses for destruction. This CRISPR/Cas system confers immunity to viruses in bacteria and archaea [30,31]. From the three types of CRISPR/Cas systems described, the type II system, as shown by Jinek et al., is capable of DNA cleavage by a single Cas protein called Cas9 [29]. This type II system utilizes two different RNA fragments a crRNA (CRISPR RNA) capable of recognizing specific DNA sites and tracrRNA (trans-acting crRNA) which hybridizes with crRNA to form a guide RNA [29]. Guide RNA and Cas9 form the CRISPR-Cas9 complex capable of precise double-strand breaks (DSBs) at three base pairs 5′ to the protospacer adjacent motif (PAM) site of the DNA [29,32]. The DSBs trigger the innate cellular machinery to repair the damaged sections either via non-homologous end joining (NHEJ) or via homology-directed repair (HDR), and both repair processes can lead to gene alterations. Non-homologous end joining is error prone and can lead to gene disruption due to insertion or deletions (indels) of a few nucleotides, whereas HDR, which normally uses homologous DNA sequences (from the other allele) as a template to repair the damaged site, can be tricked into repairing the cut with novel sequences attached to flanking sequences homologous to those on either side of the cut site [15,33]. Jinek et al. also showed that crRNA and tracrRNA can be linked together to form a single guide RNA (sgRNA) with similar guiding ability as the crRNA:tracrRNA hybrid [29]. 

Since the CRISPR-Cas9 system is RNA programmable, it removes the hurdles associated with protein engineering and validation needed in ZFNs and TALENs, and as a result, the technology has witnessed a massive increase in functional genomic studies across all the major domains of life [15,16,32,33,34]. Some recent applications of CRISPR-Cas9 include disruptions of several genes such as the pigmentation gene *yellow* in *Drosophila* [35]; genes associated with reproduction in *Anopheles gambiae* [36]; genes essential for formation of urate granule in *Bombyx mori* [2]; genes involved in olfaction in the pest species *Spodoptera littoralis* [37] and *Helicoverpa armigera* [38]; and genes involved in wing patterning and pigmentation in butterflies [39,40,41,42,43,44]. CRISPR-Cas9 has also been applied to correct genetic disorders in mice [45,46] and humans [47,48]. Further advancement of CRISPR technology using deactivated Cas9 (dCas9) has been utilized for CRISPR interference (CRISPRi) [49] and CRISPR activation (CRISPRa) [50], where Cas9 is brought to targeted places in the genome to prevent and enhance flanking gene expression, respectively, and CRISPR mediated fluorescence imaging [51] where an EGFP (Enhanced Green Fluorescent Protein) tagged Cas9 protein is guided to label chromosomes.

Here, we provide a detailed description of the CRISPR methodology we use in our lab to generate CRISPR-Cas9 somatic mutants in *Bicyclus anynana* butterflies, an emerging model system in evo-devo research [52,53]. A similar protocol for CRISPR has been recently provided for butterflies of other species [54]. Our protocol, however, is also illustrated with a video and provides a detailed description of all the steps involved. Recently, using CRISPR-Cas9, we have successfully knocked out the wing selector gene *apterousA* (*apA*), whose expression is restricted to the dorsal wing surface and when deleted leads to ventral wing patterns appearing on the dorsal surface [42]; genes of the melanin pigmentation pathway that when deleted alter both the color and the morphology of wing scales [43,44,52]; and the limb and eyespot gene *Distal-less*, which appears to be involved in a reaction–diffusion mechanism to specify eyespot centers [41]. In addition to *Distal-less*, a recent study showed that at least 186 transcripts are differentially expressed in eyespot centers [55], but their role awaits functional characterization. To illustrate how functions are assigned to butterfly genes using CRISPR, we use the gene *engrailed* as a case study. Engrailed is a segment polarity gene that is expressed in the posterior segment compartments of *B. anynana* embryos [56], in the posterior compartment and in the eyespot centers of larval wings [57], and in the gold rings of eyespots during early pupal development [58]. The function of this gene in segment formation, wing development, and eyespot development in butterflies is currently unexplored.

## 2. Experimental Design

### 2.1. Experimental Stages

**Figure 1:**
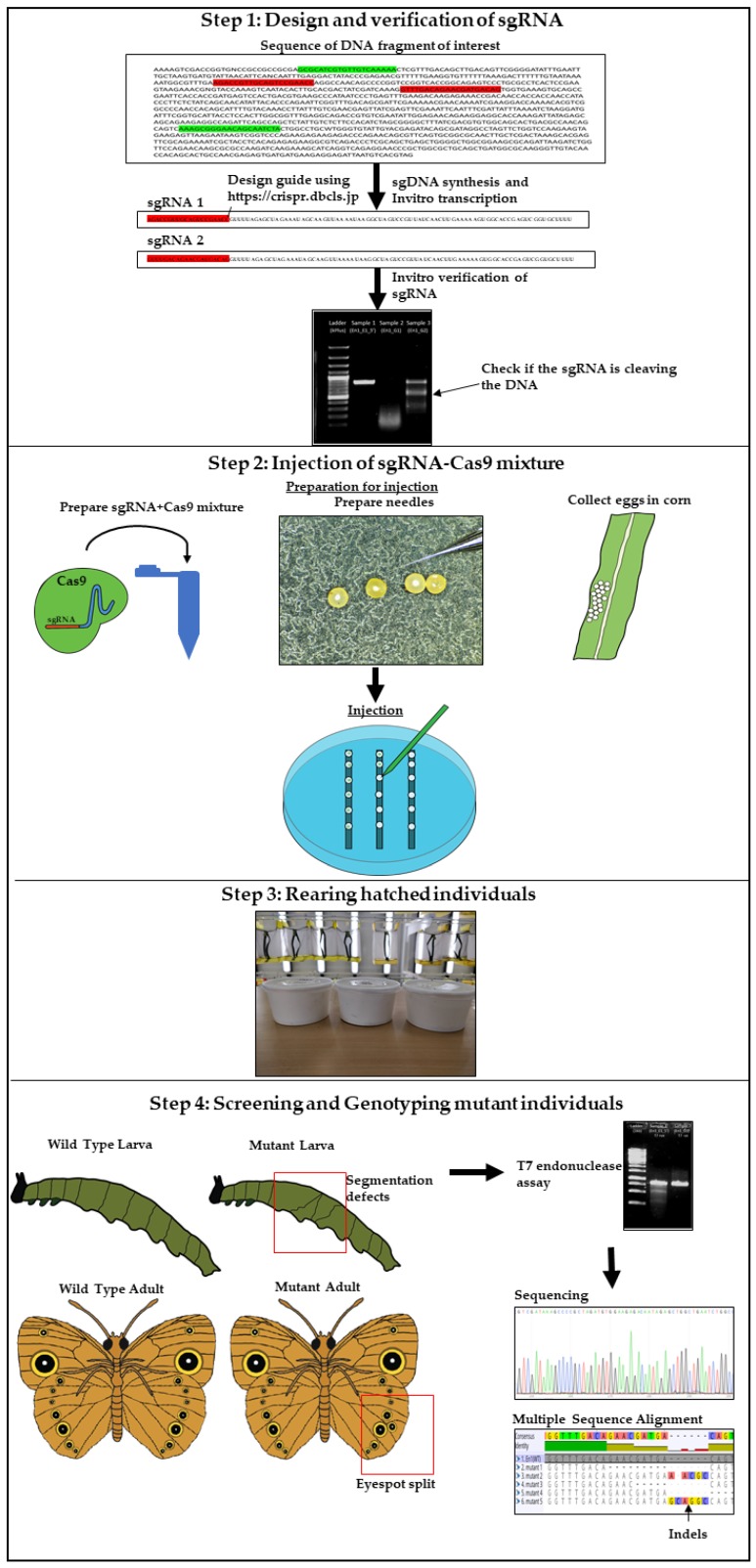
CRISPR (Clustered Regularly Interspaced Short Palindromic Repeats) workflow in *Bicyclus anynana*.

The major experimental steps involved in CRISPR-Cas9 mediated gene manipulation in *Bicyclus anynana* include: (1) Synthesis and verification of sgRNA; (2) Injection of the sgRNA-Cas9 mixture into embryos; (3) Rearing of hatched individuals, and (4) Genotyping of mutant individuals (Table 1). Figure 1 illustrates the workflow and below we provide a brief explanation of what is entailed in each of these steps. Detailed protocols then follow.

#### 2.1.1. Design, Synthesis and Verification of sgRNA

Each sgRNA contains 100 nucleotides where 20 bases contain the recognition site that mediates the hybridization with target DNA, and the remaining 80 bases are involved in sgRNA-Cas9 hybridization [59]. The 20 bases are going to be target specific, whereas the remaining 80 bases are conserved across all experiments. The sgRNA is synthesized using ultramers (long DNA sequences) with the forward primer consisting of 64 bases and reverse primer 80 bases. A double-stranded sequence of the sgRNA (sgDNA) is synthesized using high fidelity polymerase and is later used as a template for synthesizing sgRNA using in vitro transcription. After purification of sgRNA, the efficacy of sgRNA-Cas9 hybrid is verified via an in vitro cleavage assay. The presence of multiple bands in the treated sample ensures that the sgRNA is functional. 

#### 2.1.2. Injection of the sgRNA-Cas9 Mixture into Embryos

In this step, sgRNA and Cas9 protein (or mRNA) are mixed together along with Cas9 buffer and non-toxic food dye (for visualizing the amount of sgRNA-Cas9 injected). The RNA–protein hybrid can also be stored at 4 °C or −20 °C for future use. The mixture is injected using fine glass needles at optimal pressure. This step requires good injection skills and proper tools and materials to prepare fine needles (see procedure for detailed information).

#### 2.1.3. Rearing of Hatched Individuals

Rearing hatched larvae and adults requires proper humidity (80%), temperature (27 °C), and lighting (12-12 day-night cycle). Larvae are reared on young corn leaves and adults on mashed bananas.

#### 2.1.4. Genotyping of Mutant Individuals

This step involves screening of mutants. Individuals should be monitored for mosaic clones at every stage. After screening, genomic DNA is extracted from mutant tissue and the sequence of interest is amplified using polymerase chain reaction (PCR). A T7 cleavage assay is carried out on the amplified DNA to verify whether sequence variants are present in the targeted sequence. Once sequence variants are confirmed, the amplified DNA is cloned in bacteria and sequenced. The sequences are aligned, and sites of indels are identified. 

### 2.2. Required Materials and Equipment

#### 2.2.1. Materials

- Microcentrifuge tubes 1.5 mL (Eppendorf, Hamburg, Germany; Cat. no.: T9661-500EA)
- Petri plates (AIT Biotech, Singapore; Cat. no.: 800311)
- Pipette tips—1 mL, 200 µL, 10 µL (Axygen, Union City, CA, USA; Cat. nos.: 14-222-690, 14-222-812, and 14-222-737)
- 20 µL Microloader (Eppendorf; Cat. no.: 5242956003)
- Glass capillary tubes: Glass 1BBL w/FIL 1.0MM 3 IN (World Precision Instruments, Inc., Sarasota, FL, USA; Cat. no.: 1B100F-3)
- PCR tubes 200 µL (Axygen; Cat. no.: 14-222-262)
- 50 mL PYREX Round Bottom glass test tubes (Corning, Corning, NY, USA; Cat. no.: 2619H22)
- 0.5 mm stainless steel beads (Next Advance, Troy, NY, USA; Cat. no.: SKU: SSB05)
- Paper cups and cages (for rearing animals)

#### 2.2.2. Equipment

- Shaking heat block (Thermomixer, Eppendorf)
- Water bath (JULABO TW9; JULABO, Seelbach, Germany)
- Thermocycler (Super Cycler SC300G, Kyratec, Mansfield, Australia)
- Tabletop centrifuge (Eppendorf 5415 D; Eppendorf)
- Refrigerated centrifuge (Eppendorf 5810 R, Eppendorf; Hettich MIKRO 220R, Hettich ZENTRIFUGEN, Frankenberg, Germany)
- Vortex (Genie 2; Scientific Industries, Bohemia, NY, USA)
−80 °C freezer
−20 °C freezer
- 4 °C refrigerator
- Needle puller (Flaming/Brown micropipette puller, Model P-97; Sutter Instrument Co., Novato, CA, USA)
- Micro injector (Eppendorf FemtoJet 4i, Eppendorf; Parker Instrumentation Picospritzer III, Parker Instrumentation, Janesville, WI, USA)
- Sanger sequencer (3730xl; Thermo Fisher Scientific, Waltham, MA, USA)
- Pipettes: 200–1000 µL, 20–200 µL, 0.5–10 µL (Eppendorf Research or Research Plus; Eppendorf)
- Gel documentation system (Syngene GeneGenius; Syngene, Frederick, MD, USA)
- Nanodrop ND-1000 Spectrophotometer (Thermo Scientific Nanodrop ND-1000; Thermo Fisher Scientific)
- Microwave (Sharp, Osaka, Japan)
- Weighing balance (Sartorius ENTRIS623i-1S; Sartorius, Göttingen, Germany)
- Imaging system (LEICA DMS 1000; Leica Microsystems, Wetzlar, Germany)
- MilliQ water purification system (Merck Millipore, Burlington, MA, USA)
- Gel electrophoresis system (Bio-Rad, Hercules, CA, USA)
- Vacuum concentrator (Thermo Scientific SAVANT DNA120 SpeedVac Concentrator; Thermo Fisher Scientific)
- Tabletop spinner (Labnet Mini Centrifuge C1201; Labnet, Edison, NJ, USA)
- pH meter (HANNA Edge HI2020-02; HANNA, Woonsocket, RI, USA)
- Class II Biological Safety cabinet (LabCard, Lenexa, KS, USA)
- Homogenizer (Next Advance Bullet Blender; Next Advance)
- Autoclave (HIRAYAMA, Saitama, Japan)

#### 2.2.3. Reagents for sgRNA Synthesis

- Ultramers 100 mM (Sigma-Aldrich, St. Louis, MO, USA; En1_G1, En1_G2 and CRISPR_R; see Table 2 for sequence
- dNTP mix (Promega, Madison, WI, USA; Cat. no.: U1511; NEB, Ipswich, MA, USA; Cat. no.: N0447S)
- Q5 high fidelity DNA polymerase (NEB; Cat. no.: M0491S)
- Q5 polymerase buffer 10X (NEB; Cat. no.: B9027S)
- Molecular grade water (HyPure Molecular Biology Grade Water, HyClone, Thermo Fisher Scientific; Cat. no.: 7732-18-5)
- Agarose molecular grade (Vivantis, Selangor Darul Ehsan, Malaysia; Cat. no.: PC0701-500g)
- Trizma base (Sigma-Aldrich; Cat. no.: T1503-500G)
- EDTA (ThermoFisher Scientific; Cat. no.: 17892)
- Acetic acid (Sigma-Aldrich; Cat. no.: 64-19-7)
- SYBRSafe (Invitrogen, Carlsbad, CA, USA; Cat. no.: S33102)
- GeneJet PCR purification kit (Thermo Fisher Scientific; Cat. no.: K0702)
- T7 10× buffer (NEB; Cat. no.: M0251S)
- T7 RNA polymerase (NEB; Cat. no.: M0251S)
- ATP, GTP, CTP and UTP: 5 µmol (Thermo Fisher Scientific; Cat. nos.: 18330-019, 18331-017, 18332-015, and 18333-013)
- Ribolock RNase inhibitor (Thermo Fisher Scientific; Cat. no.: EO0381)
- DNA ladder (GeneDireX Kplus, Keelung City, Taiwan, Cat. No: DM011-R500/; Invitrogen 1 Kb and KbPlus DNA ladder, Thermo Fisher Scientific; Cat. nos.: 10787018 and SM0313)
- DNase I (Thermo Fisher Scientific; Cat. no.: EN0525)
- 10× DNase I buffer (Thermo Fisher Scientific; Cat. no.: AM8170G)
- NaOAc (Sigma-Aldrich; Cat. no.: S2889)
- Ethanol 100% (VWR chemicals, VWR, Radnor, PA, USA; Cat. no.: 64-17-5)
- 6× DNA loading dye (Thermo Fisher Scientific; Cat. no.: R0611)
- Riboruler High Range RNA Ladder 20 (Thermo Fisher Scientific; Cat. no.: SM1821)
- 2× RNA loading Dye (Thermo Fisher Scientific; Cat. no.: R0641)

#### 2.2.4. Reagents for sgRNA In Vitro Verification

**Figure 2:**
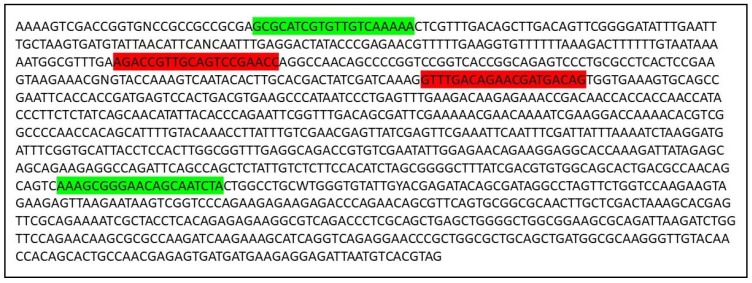
Sequence of En_1_Exon_1 along with 5′ UTR. CRISPR-Cas9 recognition site is highlighted in red and oligonucleotides for amplifying DNA are highlighted in green.

- Tissue DNA extraction Kit (E.Z.N.A Tissue DNA Kit, Omega Bio-tek, Norcross, GA, USA; Cat. no.: D3396-02)
- 2× PCRBIO Taq Mix Red (PCR Biosystems, London, UK; Cat. no.: PB10.11-20)
- Oligonucleotides 100 mM (Integrated DNA Technologies, Coralville, IA, USA; En1_E1_5′_F and En1_E1_5′_R; see Table 24 and Figure 2 for sequence)
- Molecular grade water (HyPure Molecular Biology Grade Water, HyClone, Thermo Fisher Scientific; Cat. no.: 7732-18-5)
- Cas9 Protein NLS (NEB; Cat. no.: M0641)
- Cas9 buffer 10× (NEB; Cat. no.: M0641)
- Agarose Molecular grade (Vivantis; Cat. no.: PC0701-500g)
- SYBRSafe (Invitrogen; Cat. no.: S33102)
- PCR purification kit (Thermo Fisher Scientific GeneJET PCR Purification Kit; Cat. no.: K0701)

#### 2.2.5. Reagents for CRISPR Injection

- Cas9 Protein NLS (NEB; Cat. no.: M0641)
- Cas9 buffer 10× (NEB; Cat. no.: M0641)
- Non-toxic food dye (Star Brand Artificial True Blue Color, Star Brand)
- Molecular grade water (HyPure Molecular Biology Grade Water, HyClone, Thermo Fisher Scientific; Cat. no.: 7732-18-5)

#### 2.2.6. Reagents for Isolation of Genomic DNA from Injected Embryos/Larvae/Adults

● Tissue DNA extraction kit (E.Z.N.A Tissue DNA Kit, Omega; Cat. no.: D3396-02)

#### 2.2.7. Reagents for Cloning

- Competent cells (prepared in-house; for protocol see ref. [58])
- pGEM-T Vector System (Promega; Cat. no.: A3600)
- Luria–Bertani (LB) agar (Invitrogen; Cat. no.: 22700025)
- MilliQ water (Merck Millipore)
- Ampicillin (Sigma-Aldrich; Cat. no.: 10835242001)
- Isopropyl β-d-1-thiogalactopyranoside (IPTG; Ambion, Foster City, CA, USA; Cat. no.: AM9464)
- X-Gal (Ambion; Cat. no.: 15520034)
- LB base (Invitrogen; Cat. no.: 12780029)

#### 2.2.8. Reagents for Genotyping

- Plasmid isolation kit (GeneJET Plasmid Miniprep Kit, Thermo Fisher Scientific; Cat. no.: K0502)
- Oligonucleotide (Integrated DNA Technologies; M13F and M13R; for sequence see Table 3)
- BigDye Terminator v3.1 RR-5000 and sequencing buffer (Thermo Fisher Scientific; Cat. no.: 4337457)
- PCR purification kit (Thermo Fisher Scientific GeneJET PCR Purification Kit; Cat. no.: K0701)

## 3. Procedure

### 3.1. Design, Synthesis, and Purification of sgRNA. Time for Completion: 3 Days

#### 3.1.1. Design of sgRNA

**Figure 3:**
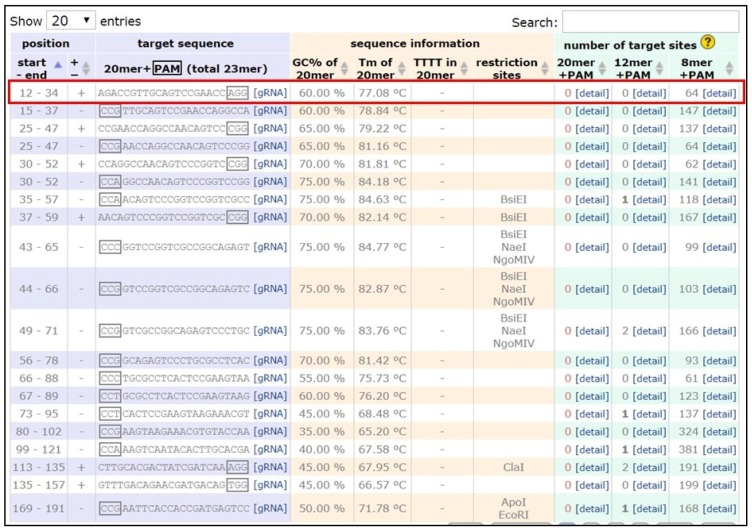
Screenshot of the guide design webpage (see ref. [59]).

1. Identify the DNA sequence to be modified. Two versions of the *B. anynana* genome are available on lepbase [60]. Genes can be searched using the search field.
2. Copy the DNA sequence and go to the webpage mentioned in [59]. Paste the sequence in the ‘Paste a nucleotide sequence’ box.
3. Apply the filter ‘Postman butterfly (*Heliconius melpomene*) genome, Hmel1 (February 2012)’ or ‘Monarch butterfly (*Danaus plexippus*) genome, DanPle_1.0 (November 2011)’ in the specificity checkbox and click the design bar.
4. Copy a candidate target sequence (see Figure 3) with orientation 5′ to 3′ (+) without the PAM sequence (Note: sequences closer to the 5′ region of the gene of interest and having high GC content should be preferred) and insert it in the grey highlighted part of the template below:
  5′-GAAATTAATACGACTCACTATAGG-xxxxxxxxxxxxxxxxxxxxx-GTTTTAGAGCTAGAAATAGC-3′
  This is the forward primer for guide synthesis.
  The reverse primer for sgRNA synthesis is the same for all target sites (see below):
  5′-AAAAGCACCGACTCGGTGCCACTTTTTCAAGTTGATAACGGACTAGCCTTATTTTAACTTG
  CTATTTCTAGCTCTAAAAC-3′.

#### 3.1.2. Synthesis of sgDNA

1. Add molecular grade water to the lyophilized primers in their original tubes (forward and reverse) to make a stock solution of 100 mM. Prepare a working solution of 10 mM by combining 10 µL of the stock solution with 90 µL molecular grade water in a new set of 1.5 mL tubes.
2. Add reagents as mentioned in Table 4 in a 200 µL PCR tube: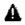 **CRITICAL STEP:** Use high fidelity DNA polymerase only. Prepare at least three tubes for each sgDNA to obtain enough yield.
3. Setup the PCR reaction with the conditions mentioned in Table 5:
4. Run the PCR reaction in 1% agarose gel.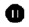 **PAUSE STEP:** The PCR reaction mixture can be stored at 4 °C overnight.

#### 3.1.3. Purification of sgDNA (Using Thermo Scientific GeneJET PCR Purification Kit)

1. Transfer the completed reaction volume to a 1.5 mL microcentrifuge tube and add an equal volume of binding buffer. Vortex the mixture for 5 s.
2. Transfer the mixture to the GeneJET PCR purification column and centrifuge at 13,000 rpm for 30 s. Discard the flow through.
3. Add 500 µL of wash buffer and centrifuge at 13,000 rpm for 30 s. Discard the flow through and repeat this step one more time.
4. Spin the moist column for one additional min at 13,000 rpm and discard the collection tube.
5. Transfer the column to a new 1.5 mL microcentrifuge tube and add 20 µL of elution buffer or molecular grade water. Incubate the column at room temperature for 3–5 min.
6. Centrifuge at 13,000 rpm for 1 min and measure the concentration of the elution using Nanodrop.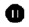 **PAUSE STEP:** Prepare a working concentration of 500 ng/µL. The purified DNA can be stored at 4 °C for over one month. For long-term storage, use a −20 °C freezer.

#### 3.1.4. In Vitro Transcription to Prepare sgRNA

1. Add the reagents mentioned in Table 6 in a 1.5 mL microcentrifuge tube:
2. Incubate the mixture in water bath at 37 °C for 16 h.
3. Add 1 µL of DNaseI and 2 µL of 10× DNaseI buffer.
4. Incubate the mixture in water bath at 37 °C for 15 min.
5. Remove 1 µL of the reaction mixture in a 200 µL PCR tube. Add 7 µL of molecular grade water and 2 µL of 2× RNA loading dye.
6. Heat the sample at 70 °C for 10 min and run it in 1% agarose gel.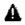 **CRITICAL STEP:** RNA degrades very fast. To prevent degradation properly, clean the gel tray using MilliQ water, use fresh buffer and run the gel for 15–20 min at low voltage.

#### 3.1.5. Purification of sgRNA (via Ethanol Precipitation)

1. Add 80 µL of molecular grade water to the reaction tube from the previous step to raise the volume to 100 µL.
2. Add 10 µL of 3 M NaOAc and 200 µL of 100% ethanol.
3. Vortex the mixture for 10 s and store at −20 °C for 15–20 min.
4. Centrifuge the mixture at 4 °C, 14,000 rpm for 15 min.
5. Carefully remove the supernatant.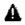 **CRITICAL STEP:** Be very careful not to disturb the pellet.
6. Dry the sample in a vacuum concentrator and add 20 µL of molecular grade water.
7. Prepare a stock concentration of 600 ng/µL by adding additional water (after a Nanodrop reading) and store aliquots at −20 °C.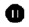 **PAUSE STEP:** RNA can be stored at −20 °C for over 1 year.

### 3.2. OPTIONAL STEP: Preparation and Purification of Cas9 mRNA. Time for Completion: 2 Days

#### 3.2.1. Preparation of Cas9 mRNA (Using mMESSAGE mMACHINE T3 Kit and Poly(A) Tailing Kit, ThermoFisher Scientific)

1. Add 10 µg of Addgene plasmid #46757 (pT3TS-nCas9n), 2 µL of restriction enzyme and molecular grade water (to make up the volume to 20 µL) in a 1.5 mL microcentrifuge tube.
2. Incubate the mixture at 37 °C for 2 h.
3. Add the reagents mentioned in Table 7 in a 1.5 mL centrifuge tube:
4. Incubate the mixture in water bath at 37 °C for 4 h.
5. Add 1 µL of DNase I and 2 µL of 10× DNase buffer and incubate in water bath at 37 °C for 15 min.
6. For poly(A) tailing add the reagents mentioned in Table 8 to the tube above:
7. Incubate the mixture in water bath at 37 °C for 1 h.

#### 3.2.2. Purification of Cas9 mRNA

1. Add 60 µL of LiCl_2_ (provided in the mMESSAGE mMACHINE kit) to the tube above and incubate at −20 °C for 30 min.
2. Centrifuge at 4 °C, 14,000 rpm for 15 min.
3. Carefully remove the supernatant and resuspend the pellet in 100 µL 70% ethanol.
4. Centrifuge at 4 °C, 14,000 rpm for 15 min.
5. Remove the supernatant and dry the sample in a vacuum concentrator.
6. Resuspend the pellet in 10 µL molecular grade water and measure the concentration using Nanodrop. Store the RNA at −20 °C.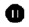 **PAUSE STEP:** RNA can be stored at −20 °C for over one year.

### 3.3. Isolation of the Fragment of Interest (e.g., from Genomic DNA, Plasmid, etc.) and Verification of sgRNA via Invitro Cleavage. Time for Completion: 1 Day

#### 3.3.1. Isolation of Genomic DNA (Using E.Z.N.A Tissue DNA Kit, Omega Bio-tek)

1. Remove the epidermis of 5th instar larvae and transfer the tissue into a 1.5 mL microcentrifuge tube.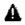 **CRITICAL STEP:** Carefully remove the gut material from the larvae as it might contaminate the DNA sample. One larva should be enough for a yield of around 500 ng/µL in 100 µL volume. Alternatively, tissues can be extracted from the thorax of adult *Bicyclus* or embryos.
2. Add 200 µL TL buffer and homogenize the tissue in homogenizer using 0.5 mm stainless steel beads for 5 min.
3. Add 20 µL OB protease solution and vortex for 10 s.
4. Incubate the mixture in shaking heat block at 55 °C for 16 h.
5. Centrifuge the tube at 14,000 rpm for 5 min to precipitate the cell debris.
6. Transfer the supernatant to a fresh 1.5 mL microcentrifuge tube and add 220 µL BL buffer.
7. Incubate the mixture in water bath at 70 °C.
8. Add 220 µL 100% ethanol and vortex for 10 s.
9. Transfer the mixture to HiBind DNA column and centrifuge at 14,000 rpm for 1 min.
10. Discard the filtrate and add 500 µL HBC buffer.
11. Centrifuge at 14,000 rpm for 30 s and discard the filtrate.
12. Transfer the column to a fresh 2.0 mL collection tube.
13. Add 500 µL DNA wash buffer and centrifuge at 14,000 rpm for 30 s. Discard the flow through and repeat this step one more time.
14. Centrifuge the empty column at 14,000 rpm for 1 min and transfer the column into a fresh 1.5 mL microcentrifuge tube.
15. Add 100 µL elution buffer or molecular grade water to the column and let it sit for 5 min at room temperature.
16. Centrifuge at 14,000 rpm for 1 min and measure the concentration using Nanodrop. Store the DNA at 4 °C for immediate use.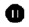 **PAUSE STEP:** DNA can be stored at −20 °C for over three years.

#### 3.3.2. Design of Primers for Amplification of DNA Fragment of Interest.

1. Copy the sequence of interest and paste it into the box on the webpage mentioned in ref [61].
2. Under the ‘General Setting’ tab change the values:
  Primer Tm: Min: 55; Max: 65
  Primer GC%: Min: 45; Opt: 60; Max: 60
  Max Tm Difference: 3
3. Click on the ‘Pick Primers’ tab in the top right corner.
4. Select the best set from the list of primers.

#### 3.3.3. Amplification of DNA Fragment of Interest

1. Resuspend the lyophilized primers using molecular grade water to make a stock solution of 100 ng/µL. Prepare a working solution of 10 mM as described above.
2. Add the reagents mentioned in Table 9 in a 200 µL PCR tube: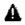 **CRITICAL STEP:** Prepare at least five tubes in order to identity the most optimal annealing temperature in a gradient PCR reaction.
3. Setup the gradient PCR reaction with conditions as mentioned in Table 10:
4. Run the reaction mixture in 1% agarose gel for 30 min.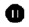 **PAUSE STEP:** The PCR reaction mixture can be stored at 4 °C overnight.

#### 3.3.4. Purification of Amplified DNA (Using ThermoFisher Scientific GeneJET PCR Purification Kit)

1. Transfer the reaction volume to a 1.5 mL microcentrifuge tube and add an equal volume of binding buffer. Vortex the mixture for 5 s.
2. Transfer the mixture to the GeneJET PCR purification column and centrifuge at 13,000 rpm for 30 s. Discard the flow through.
3. Add 500 µL of wash buffer and centrifuge at 13,000 rpm for 30 s. Discard the flow through and repeat this step one more time.
4. Spin the moist column for and additional min at 13,000 rpm and discard the collection tube.
5. Transfer the column to a new 1.5 mL microcentrifuge tube and add 20 µL of elution buffer or molecular grade water. Incubate the column at room temperature for 3–5 min.
6. Centrifuge at 13,000 rpm for 1 min and measure the concentration using Nanodrop.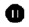 **PAUSE STEP:** The purified DNA can be stored at 4 °C for over one month. For long-term storage use a −20 °C freezer.

#### 3.3.5. Verification of sgRNA via In Vitro Cleavage

1. Add the reagents mentioned in Table 11 in a 1.5 mL microcentrifuge tube:
2. Incubate the sample in a water bath at 37 °C for 10 min.
3. Add 200 ng of the amplified DNA of interest with the recognition site and incubate the mixture in the water bath at 37 °C for 20 min.
4. Run 5 µL of the mixture along with 100 ng of the original template DNA in 1% agarose gel for 30 min.

#### 3.3.6. OPTIONAL STEP: Alternative Verification of sgRNA (If Step 3.3.5 Does Not Work)

1. Add the reagents mentioned in Table 12 in a 1.5 mL microcentrifuge tube:
2. Add 0.1 µL of Cas9 Protein NLS (3300 ng/µL) and incubate the mixture at 25 °C for 10 min.
3. Add 100 µg/mL BSA (Bovine Serum Albumin) and incubate the mixture in a water bath at 37 °C for 3 h.
4. Add 1 µL of proteinase K and incubate the mixture in a water bath at 37 °C for 10 min.

### 3.4. Embryo Collection, Needle Preparation, and Injection of the sgRNA-Cas9 Mixture: Time for Completion: 1 Day

#### 3.4.1. Needle Preparation

**Figure 4:**
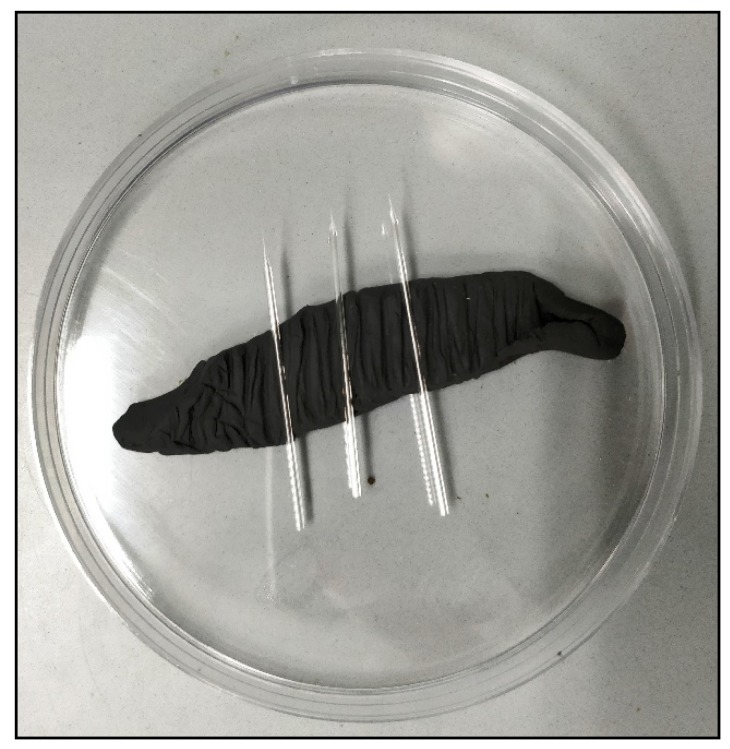
Needles on plasticine.

1. Turn on the needle puller machine and set a program with the settings as mentioned in Table 13:
2. Arrest the glass capillary in place and click on the enter button. Wait for the process of heating and pulling the glass to be completed.
3. Carefully remove the needles from the machine and place them on plasticine (as shown in Figure 4).

#### 3.4.2. Embryo Collection

**Figure 5:**
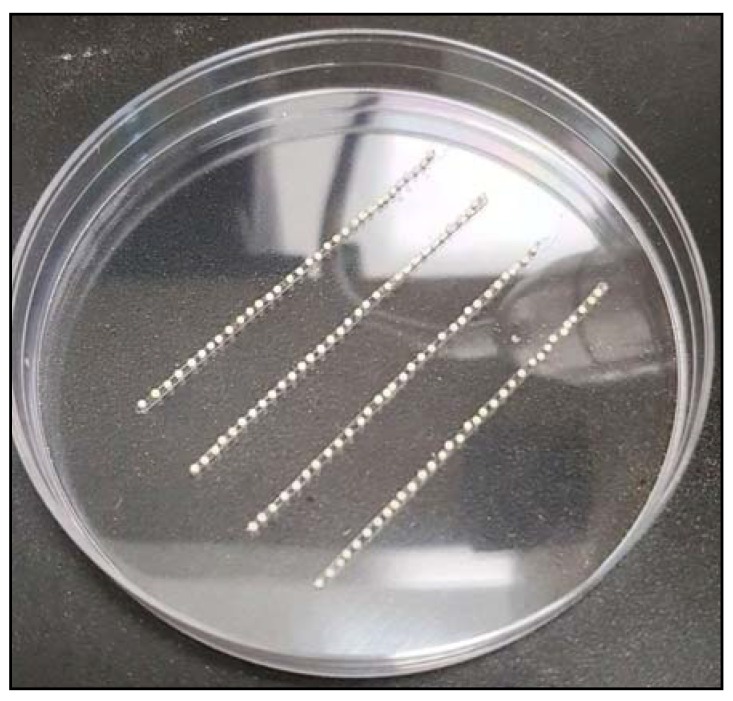
Embryos on double-sided tape.

1. Place a few young corn leaves inside butterfly cages and leave them for 30 min. (Note: The best time for embryo collection is around 2:00 p.m. to 3:00 p.m.).
2. Remove the leaves and collect the embryos in a paper cup.
3. Prepare a Petri dish with thin strips of double-sided tape attached to the bottom of the plate.
4. Using a paintbrush, carefully arrange the embryos on the double-sided tape (Figure 5).

#### 3.4.3. Preparation of sgRNA-Cas9 Mixture

1. Add the reagents mentioned in Table 14 in a 1.5 mL microcentrifuge tube:
2. Incubate the mixture in a water bath at 37 °C for 10 min.
3. Add 0.5 µL non-toxic food dye and store at room temperature until use.

#### 3.4.4. Injection of the sgRNA-Cas9 Mixture into Embryos

1. Turn on the injector and set the conditions as follows:
  For PICOSPRITZER III
  DURATION: 30 milliseconds
  For FemtoJet 4i
  pi[PSI] = 0.50; pc[PSI] = 0.10 for t[sec] = 0.50
2. Pipette 3 µL of the sgRNA-Cas9 mix using a 20 µL Microloader tip and transfer the content to the needle by filling it from the back.
3. Attach the needle to the injection holder and break the tip of the needle (e.g., remove the molten glass at the tip that obstructs the opening) by gently pressing against the side of a Petri dish under a dissecting microscope.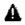 **CRITICAL STEP:** Be careful while breaking the needle tip. If the desired sharpness is not attained, transfer the mixture back to the 1.5 mL tube (by pushing it out with air pressure) and repeat the steps above.
4. Inject the embryos under a dissecting microscope until the mixture is visible inside the embryos.

### 3.5. Rearing Hatchling and Screening for Mutants. Time for Completion: 4–5 Weeks

#### Rearing of the Hatchlings (Condition for Rearing Include 12-12 Day-to-Night Cycle, Temperature of 27 °C, and Humidity of 80%).

1. Place a wet cotton ball inside the Petri plate above and incubate at 27 °C. It will take 3–4 days for the embryos to hatch (use the unhatched embryos for the T7 endonuclease assay).
2. Transfer the hatched larvae into a paper cup with one young corn leaf. Keep feeding these larvae inside cups with fresh cut leaves until they reach the 3rd instar. Transfer these older larvae to larger rearing cages. Keep note of any changes in phenotype and image these abnormal individuals/tissues.
3. Pupae are assigned to separate paper or plastic cups, individually, where adults emerge after one week.
4. Freeze the adults at −20 °C for imaging and genotyping.

### 3.6. T7 Endonuclease Assay and Genotyping of Mutants. Time for Completion: 3 Days

#### 3.6.1. Isolation of Genomic DNA from Mutant Tissue (using E.Z.N.A Tissue DNA Kit)

1. Remove mutant clones of cells (such as epidermis of larvae with segmentation defect, wing region with extra eyespot etc.) and transfer into a 1.5 mL microcentrifuge tube.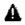 **CRITICAL STEP:** Carefully remove the gut material from the larvae to minimize contaminating the larval DNA with bacterial DNA.
2. Add 200 µL TL buffer and homogenize the tissue in homogenizer using 0.5 mm stainless steel beads for 5 min.
3. Add 20 µL OB protease solution and vortex for 10 s.
4. Incubate the mixture in a shaking heat block at 55 °C for 16 h.
5. Centrifuge the tube at 14,000 rpm for 5 min to precipitate the cell debris.
6. Transfer the supernatant to a fresh 1.5 mL microcentrifuge tube and add 220 µL BL buffer.
7. Incubate the mixture in a water bath at 70 °C.
8. Add 220 µL of 100% ethanol and vortex for 10 s.
9. Transfer the mixture to a HiBind DNA column and centrifuge at 14,000 rpm for 1 min.
10. Discard the filtrate and add 500 µL of HBC buffer.
11. Centrifuge at 14,000 rpm for 30 s and discard the filtrate.
12. Transfer the column to a fresh 2.0 mL collection tube.
13. Add 500 µL DNA wash buffer and centrifuge at 14,000 rpm for 30 s. Discard the flow through and repeat this step one more time.
14. Centrifuge the empty column at 14,000 rpm for 1 min and transfer the column into a fresh 1.5 mL microcentrifuge tube.
15. Add 100 µL elution buffer or molecular grade water to the column and let it sit for 5 min at room temperature.
16. Centrifuge at 14,000 rpm for 1 min and measure the concentration using Nanodrop. Store the DNA at 4 °C for immediate use.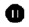 **PAUSE STEP:** DNA can be stored at −20 °C for over three years.

#### 3.6.2. Amplification of DNA Fragment of Interest from DNA Isolated from Mutant Tissue

1. Add the reagents mentioned in Table 15 in a 200 µL PCR tube: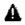 **CRITICAL STEP:** Prepare at least five tubes to identify the most optimal annealing temperature using gradient PCR.
2. Setup the PCR reaction with the conditions mentioned in Table 16:
3. Run the reaction mixture in 1% agarose gel for 30 min.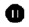 **PAUSE STEP:** The PCR reaction mixture can be stored at 4 °C overnight.

#### 3.6.3. Purification of Amplified DNA (Using ThermoFisher Scientific GeneJET PCR Purification Kit)

1. Transfer the completed reaction volume to a 1.5 mL microcentrifuge tube and add an equal volume of binding buffer. Vortex the mixture for 5 s.
2. Transfer the mixture to the GeneJET PCR purification column and centrifuge at 13,000 rpm for 30 s. Discard the flow through.
3. Add 500 µL of wash buffer and centrifuge at 13,000 rpm for 30 s. Discard the flow through and repeat this step one more time.
4. Centrifuge the empty column for one additional min at 13,000 rpm and discard the collection tube.
5. Transfer the column to a fresh 1.5 mL microcentrifuge tube and add 20 µL of elution buffer or molecular grade water. Incubate the column at room temperature for 3–5 min.
6. Centrifuge at 13,000 rpm for 1 min and measure the concentration using Nanodrop.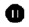 **PAUSE STEP:** Prepare a working concentration of 200 ng/µL. The purified DNA can be stored at 4 °C for over one month. For long-term storage use a −20 °C freezer.

#### 3.6.4. T7 Endonuclease Assay on Amplified DNA Fragments

1. Prepare two 200 µL PCR tubes and add the reagents mentioned in Table 17 into each of them:
2. Perform T7 hybridization in thermocycler with the conditions mentioned in Table 18:
3. Add 1 µL T7 endonuclease in one tube and incubate both tubes in water at 37 °C for 15 min.
4. Run the samples in 1% agarose gel for 30 min.

#### 3.6.5. Cloning of amplified DNA fragments (using pGEM-T Vector System)

1. Add the reagents mentioned in Table 19 in a 1.5 mL microcentrifuge tube (ligation mixture):
2. Incubate the reaction mixture at 4 °C for 16 h.
3. Take out one vial of competent cells and keep the tube on ice for 15 min.
4. Transfer 5 µL of ligation mixture into the competent cell tube and tap gently to mix the solution.
5. Leave the mixture on ice for 30 min.
6. Heat shock the cells by transferring the tube into a water bath at 42 °C for 45 s.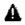 **CRITICAL STEP:** Be careful not to exceed the heat shock step above 45 s.
7. Transfer the tube into ice and leave it for 2 min.
8. Add 500 µL of autoclaved LB broth and incubate the cells in bacterial incubation chamber at 37 °C with shaking speed of 225 rpm for 2 h.
9. Centrifuge the tube at 3000 rpm for 4 min.
10. Inside a biological safety cabinet add the reagents mentioned in Table 20 to an LB agar plate:
11. Spread the reagents on the plate using glass beads and let the plate dry inside the hood.
12. Add 50 µL of supernatant from step 9 and spread across the plate using the glass beads.
13. Once dried, seal the plate using parafilm and incubate the plate inside a bacterial incubator at 37 °C for 14 h.

#### 3.6.6. Colony PCR on Transformed Clones

1. In a 1.5 mL microcentrifuge tube, add 10 µL molecular grade water. Pick a transformed white colony and transfer it into the tube. Vortex gently to homogenize the colony.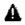 **CRITICAL STEP:** Prepare at least 10 clones (colonies) for testing.
2. Add the reagents mentioned in Table 21 in 200 µL PCR tubes:
3. Setup the PCR reaction with the conditions mentioned in Table 22:
4. Run the reaction mixture in a 1% agarose gel for 30 min and note down the colonies with a single band of the expected size, e.g., those that don’t have an empty plasmid.
5. Inside a laminar hood, add 5 µL of ampicillin stock solution into a test tube with 5 mL LB broth. Transfer 5 µLs of homogenized cells from step 1. Do this step for every positive colony.
6. Incubate the tubes in a bacterial incubation chamber at 37 °C and 225 rpm for 14–16 h.

#### 3.6.7. Isolation of Plasmids from Transformed Clones (Using GeneJET Plasmid Miniprep Kit)

1. Harvest the cells in a 1.5 mL centrifuge tube at 3000 rpm for 5 min (pellet can be stored in 40% glycerol at −80 °C for future use).
2. Discard the supernatant and resuspend the pellet in 250 µL of resuspension buffer.
3. Add 250 µL of lysis buffer and mix by inverting the tube 6–10 times.
4. Add 350 µL of neutralization buffer and mix by inverting the tube 6–10 times.
5. Centrifuge at 14,000 rpm for 5 min and transfer the supernatant to GeneJET spin column.
6. Centrifuge the column at 14,000 rpm for 30 s.
7. Add 500 µL of wash buffer and centrifuge at 14,000 rpm for 30 s. Discard the flow through and repeat this step one more time.
8. Centrifuge the empty column at 14,000 rpm for 1 min.
9. Transfer the column to a 1.5 mL microcentrifuge tube and add 20 µL of elution buffer or molecular grade water. Incubate the mixture at room temperature for 3 min.
10. Centrifuge the column at 14,000 rpm for 1 min and measure the concentration of plasmid using Nanodrop.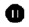 **PAUSE STEP:** Prepare a working concentration of 100 ng/µL. The purified plasmid can be stored at 4 °C for over one month. For long-term storage, use a −20 °C freezer.

#### 3.6.8. Sequencing of Cloned DNA Fragments

1. Add the reagents mentioned in Table 23 in a 200 µL PCR tube:
  Add the reagents mentioned in Table 24 in another 200 µL PCR tube:
2. Setup sequencing PCR reaction with the conditions mentioned in Table 25:
3. In 1.5 mL microcentrifuge tubes, add 40 µL molecular grade water and transfer the reaction mix from the previous step.
4. Vortex the mix and incubate at −20 °C for 20 min.
5. Centrifuge at 4 °C, 14,000 rpm for 15 min.
6. Remove the supernatant and resuspend the pellet in 100 µL 70% ethanol.
7. Centrifuge at 4 °C, 14,000 rpm for 15 min.
8. Carefully remove the supernatant and dry the sample in a vacuum concentrator.
9. Store at −20 °C until sequencing.

#### 3.6.9. Analyzing the Sequencing Results and Determination of Indel Sites

1. Copy the sequences in a word file with specific identifiers.
2. Align the sequences along with the original DNA sequence using ClustalW [63] or the Geneious multiple sequence alignment tool.
3. Look for the indels at the expected site and save the file for future use.

## 4. Expected Results

The results are explained with reference to the gene Engrailed 1 (En1). 

**Figure 6:**
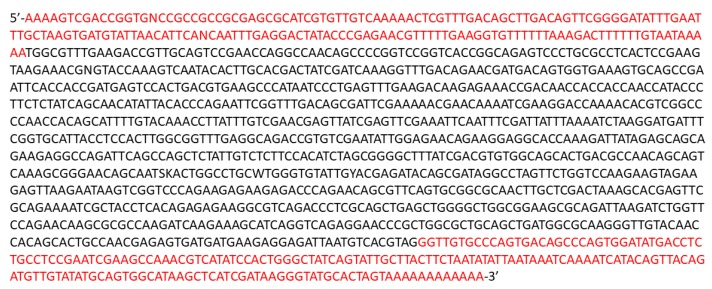
Complete transcript of En1.

The sequence of the complete transcript is given below (Figure 6) where bases in red represent the 5′ and 3′ UTRs, respectively:

### 4.1. Design, Synthesis and Purification of sgRNA

#### 4.1.1. Yield of sgDNA

Yield of sgDNA obtained for the two guides (G1 and G2): En1_G1 = 7.59 µg; En1_G2 = 6.99 µg. The expected yield of sgDNA is around 6–15 µg.

#### 4.1.2. Gel Electrophoresis of sgDNA

**Figure 7:**
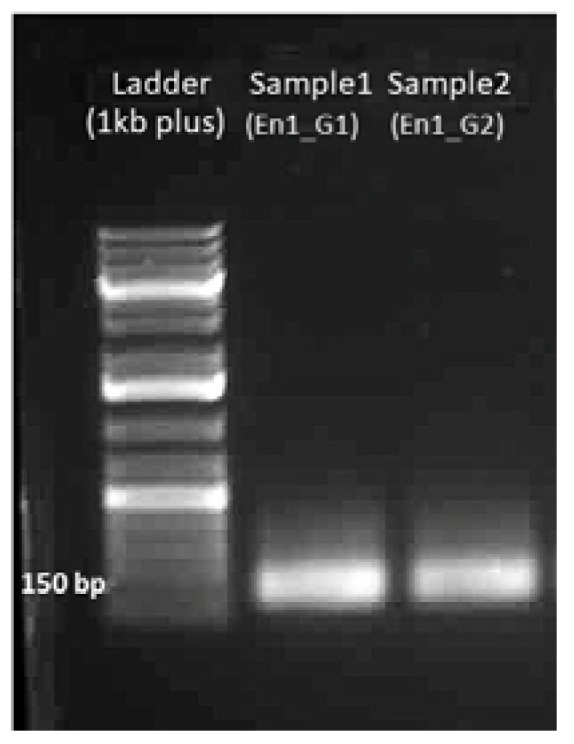
Gel electrophoresis image of sgDNA.

A band at 150 bp should be visible with 100–200 ng of the sgDNA product (see Figure 7). 

#### 4.1.3. Yield of sgRNA

Yield of sgRNA obtained: En1_G1 = 18 µg; En1_G2 = 14 µg. The expected yield of sgRNA is around 15–20 µg.

#### 4.1.4. Gel Electrophoresis of sgRNA 

**Figure 8:**
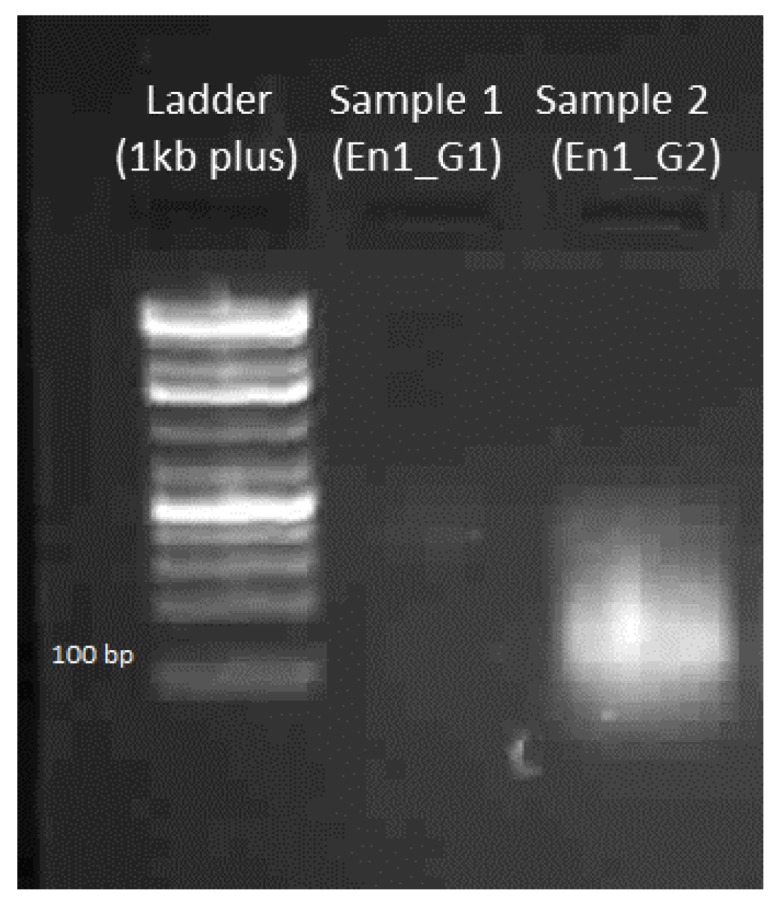
Gel electrophoresis of sgRNA: En1_G1 and En1_G2. (Note: En1_G1 is degraded possibly due to RNase contamination).

A band at 100 bp should be visible with 500 ng of the sgRNA product (see Figure 8). 

TROUBLESHOOTING: Make sure that all the tubes and pipette tips are RNase free. Use an RNase blaster solution to remove RNase contamination.

### 4.2. Preparation and Purification of Cas9 mRNA

Yield of Cas9 mRNA. The expected yield of Cas9 mRNA after purification should be around 15–40 µg. The concentration of Cas9 mRNA obtained in this experiment was 15 µg.

### 4.3. Isolation of the Fragment of Interest (e.g., from Genomic DNA, Plasmid, etc.) and Verification of sgRNA via In Vitro Cleavage

#### 4.3.1. Yield of Genomic DNA

The expected yield of genomic DNA from the epidermal tissue of one 5th instar larvae is around 50 µg. 

#### 4.3.2. Gel Electrophoresis of Amplified DNA 

**Figure 9:**
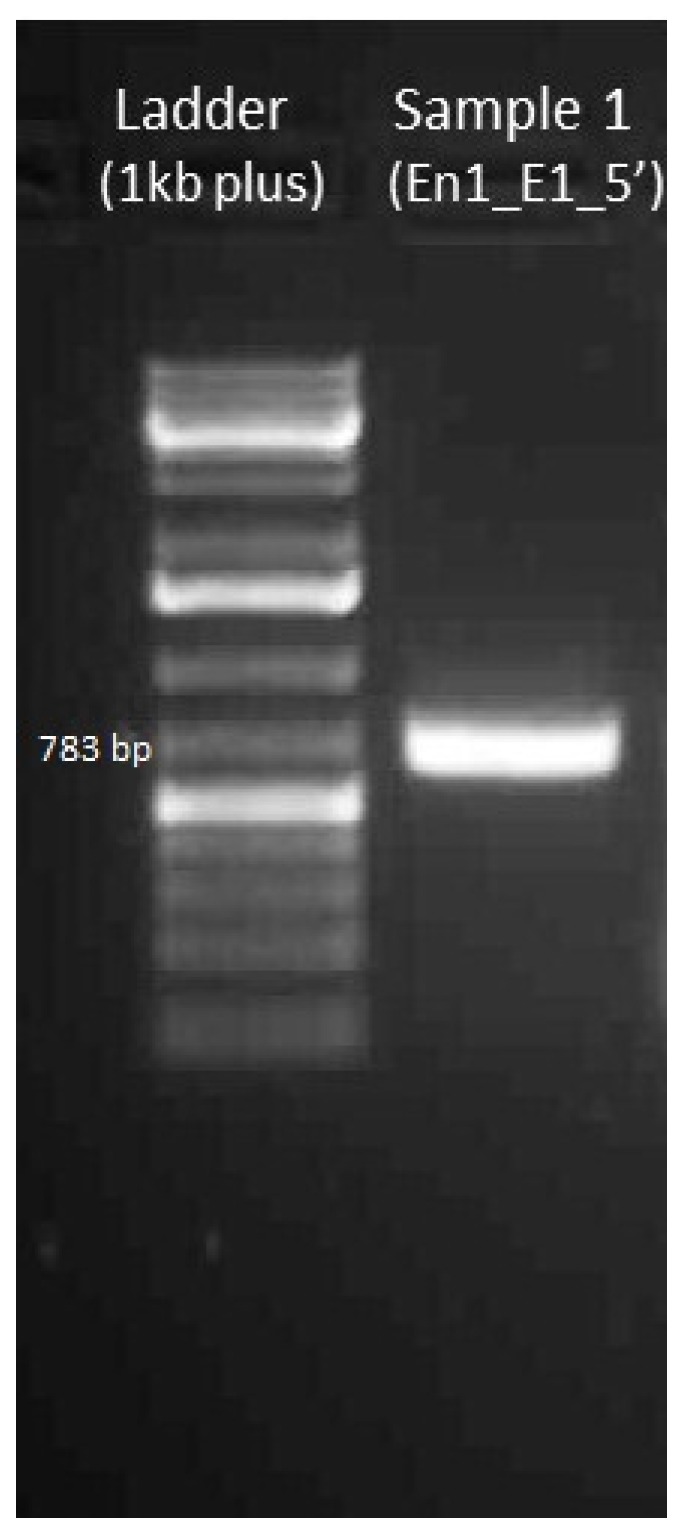
Gel electrophoresis image of the amplified DNA (En1_E1_5′).

Band at 783 bp should be visible with 100–200 ng of the PCR product with En1_E1_5′_F and En1_E1_5′_R primers (see Figure 9). 

#### 4.3.3. Yield of Amplicon

The yield of amplified DNA from 5 tubes of PCR reaction mixture was 5.6 µg. The yield usually varies in between 4 and 10 µg. 

#### 4.3.4. Gel Electrophoresis of sgRNA-Cas9 Cleaved DNA Fragment 

**Figure 10:**
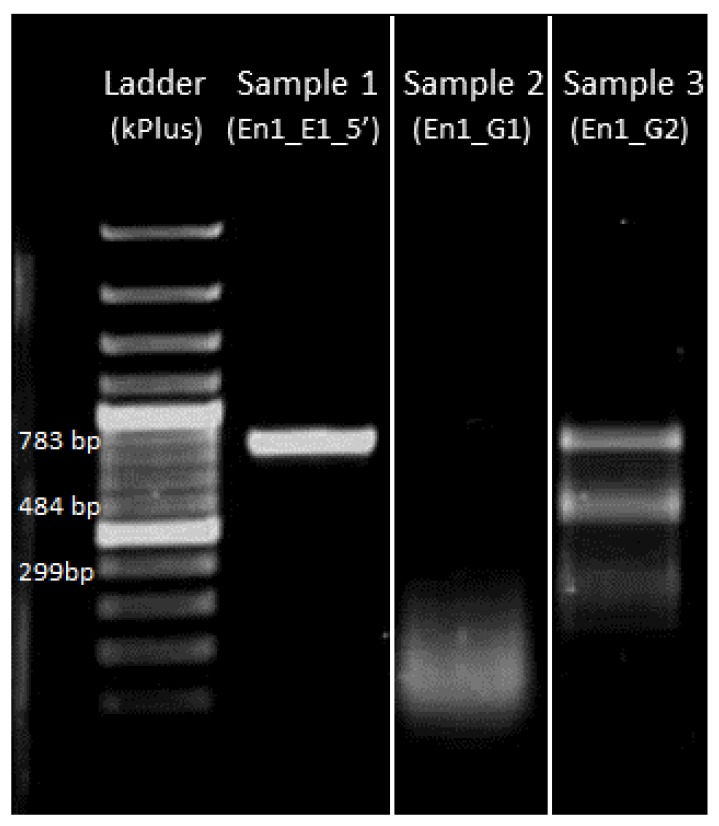
In vitro verification that sgRNA cuts the target sequence. Sample 1: Template DNA; Sample 2: Template DNA cleaved with sgRNA (En1_G1) *; Sample 3: Template DNA partially cleaved with sgRNA (En1_G2) into two smaller fragments. * Note: Reaction mixture in sample 2 was previously shown to have no sgRNA (Figure 7, Sample 1) and here the DNA smear represents degraded DNA possibly due to DNase contamination.

The cleavage assay should yield two smaller bands (see Figure 10 lane 4). The size of smaller bands denotes the site of cleavage and can be cross-verified by looking at the original template sequence and the cas9 cleavage site originally designed (see Figure 2).

### 4.4. Embryo Collection, Needle Preparation, and Injection of the sgRNA-Cas9 Mixture

#### 4.4.1. Number of Embryos

The expected number of embryos that can be obtained from 25 mated adult females is around 150–200 every 30 min. 

#### 4.4.2. Thickness of Needle

**Figure 11:**
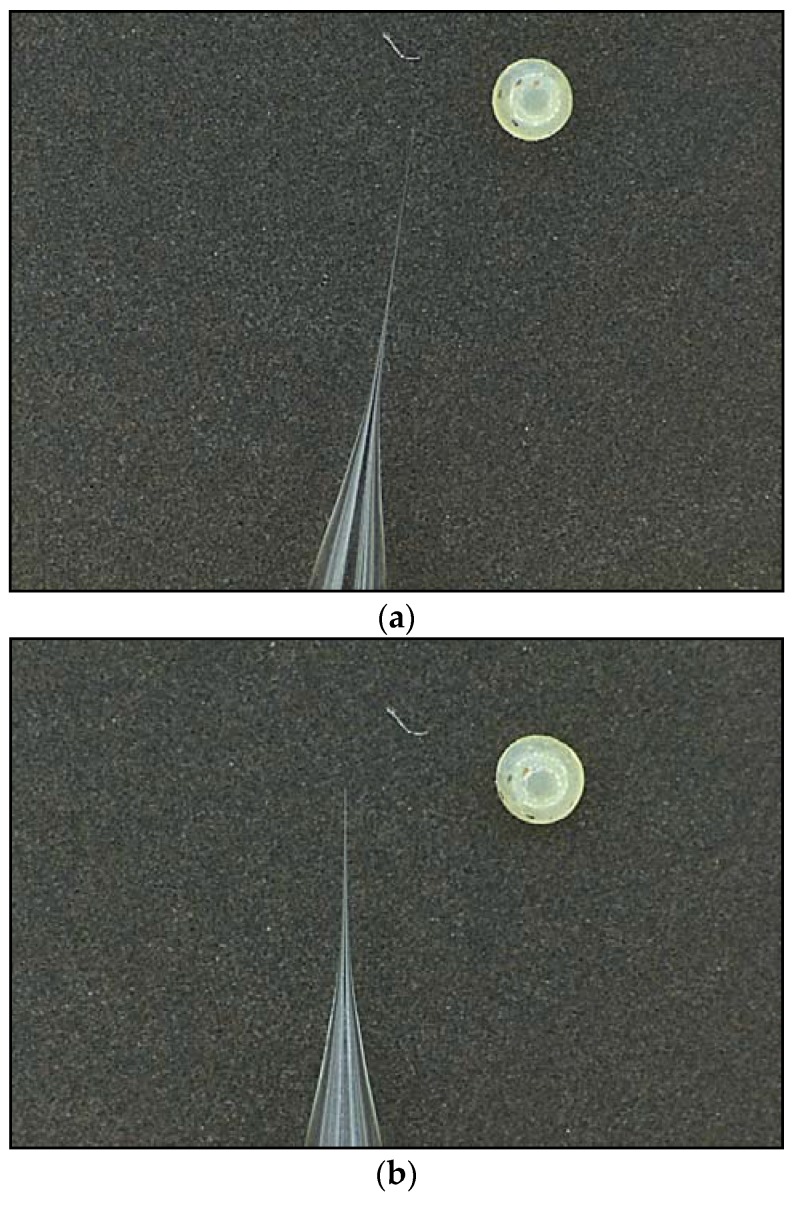
(**a**) Thickness of needle tip after pulling (*Bicyclus anynana* embryos are shown for size comparison); (**b**) thickness of needle tip after tip being broken.

The expected thickness of needle after pulling is shown in Figure 11. After breaking the tip, the diameter should be approximately 0.01–0.05 mm.

#### 4.4.3. Hatching Rate

The hatching rate usually depends on the gene targeted. For En1, the hatch rate was between 5% and 50%. Table 26 shows the number of hatched individuals using 100 ng/µL and 300 ng/µL of En1_G2 sgRNA.

#### 4.4.4. Number of Mutants

**Figure 12:**
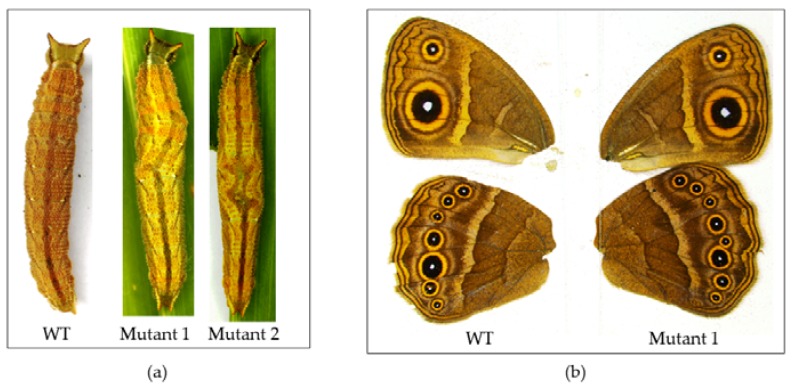
Mutant phenotypes. (**a**) 5th instar with segmentation defects; (**b**) adult phenotypes with one extra vein between Cu1 and M3 (Comstock–Needham system [59]) and splitting of the Cu1 eyespot. WT: Wild type.

In experiments 1–4, we were unable to obtain any mutants. In experiment 5, we observed six individuals during the larval stage with segmentation defects and three adult individuals with eyespot and venation defects (Figure 12).

### 4.5. T7 Endonuclease Assay and Genotyping of Mutants

#### 4.5.1. Yield of DNA from Mutant Clones

The yield of DNA from 1 larva was 2.4 µg. Expected yield is around 2–4 µg (total yield) depending on the amount of tissue used for isolation.

#### 4.5.2. Yield of DNA Amplicon

The expected yield of amplified DNA is around 4 µg.

#### 4.5.3. Gel Electrophoresis after T7 Endonuclease Assay 

**Figure 13:**
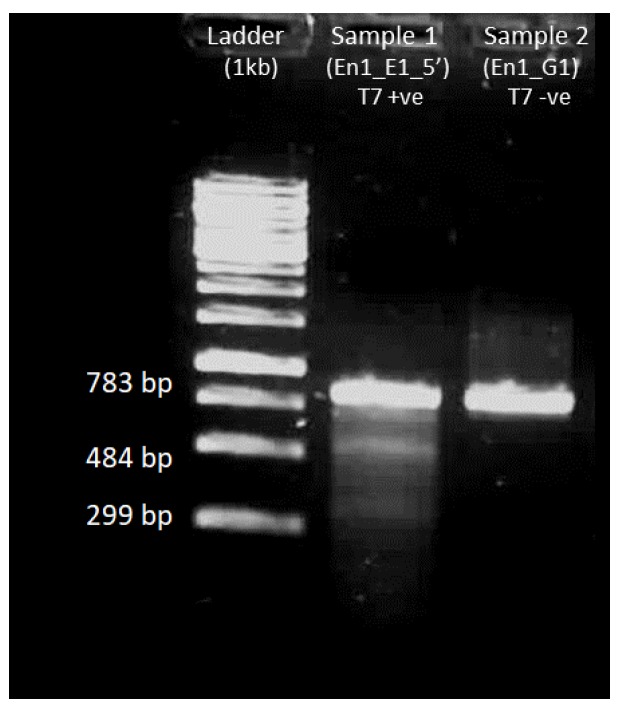
T7 endonuclease assay on amplified DNA of interest obtained from injected embryos that did not hatch. Ladder: 1 kb from Thermo Fisher Scientific; Sample 1: En1_E1_5′ with T7 endonuclease added; Sample 2: En1_E1_5′ without T7 endonuclease. Sample 1 with T7 endonuclease added shows cleavage of DNA with bands similar to the ones seen in the in vitro verification step (Figure 10 sample 3).

The T7 endonuclease assay (Figure 13) should yield smaller bands equivalent to the cleavage assay shown in Figure 10. 

#### 4.5.4. Number of Colonies

We obtained around 200 blue and white colonies after plating 50 µL of bacterial supernatant cultured for 2 h. Expected number of colonies is between 100 and 300.

#### 4.5.5. Yield of Plasmid

From 2 mL of bacterial culture grown overnight, we obtained 2.4 µg plasmid. The yield varies depending on the amount of culture used (usually between 2 and 4 µg).

#### 4.5.6. Sequencing Alignment Result

**Figure 14:**
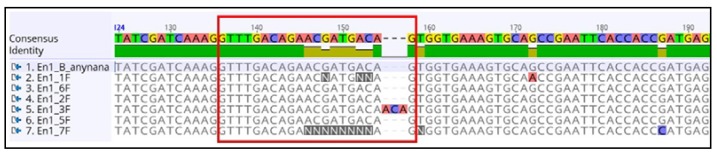
Insertion of nucleotides in the target site (sequence 1 is from the wild type). The sgRNA recognition sequence is marked by the red box.

The sites of indels can be identified by aligning the sequences and analyzing the expected target site of CRISPR-Cas9 (Figure 14). 

## 5. Reagents Setup

### 5.1. For sgRNA Design and In Vitro Verification

#### 5.1.1. Preparation of 50× TAE buffer (1 L)

1. In a 2 L beaker, add 500 mL MilliQ water and the reagents specified in Table 27.
2. Transfer the content to a 1 L measuring cylinder. Raise the volume to one liter using MilliQ water.
3. Mix the solution and transfer the content to a 1 L glass bottle.
4. Autoclave the solution at 121 °C for 20 min and store the content at room temperature.
  Note: To prepare 1× TAE, add 20 mL of 50× TAE buffer and 980 mL of MilliQ water.

#### 5.1.2. Preparation of 1% Agarose Gel

1. Weigh 0.5 g of molecular grade agarose and transfer the content to a 200 mL glass bottle.
2. Add 50 mL of 1× TAE buffer and heat the content inside microwave for 2 min.
3. Remove the bottle and add 2 µL of SYBRSafe.
4. Mix the content and pour on the gel tray.

#### 5.1.3. Preparation of 3 M NaOAc (100 mL)

1. Add 24.6 g of NaOAc in 60 mL MilliQ water.
2. Adjust the pH using 1 N NaOH or 1N HCl.
3. Transfer the content to a measuring cylinder and raise the volume to 100 mL with MilliQ.
4. Store the content in a 200 mL glass bottle at room temperature.

#### 5.1.4. Preparation of 70% Ethanol (100 mL)

1. Add 30 mL of 100% ethanol into a 200 mL measuring cylinder and raise the volume to 100 mL using MilliQ water.
2. Store the content in 200 mL glass bottle at room temperature.

### 5.2. For Cloning of Amplified DNA Fragments

#### 5.2.1. Preparation of Ampicillin (100 mM)

1. In a 20 mL measuring cylinder add 0.371 g of ampicillin and add molecular grade water up to a volume of 10 mL.
2. Mix the content and make 1 mL aliquots in 1.5 mL microcentrifuge tubes. Store the content at −20 °C.

#### 5.2.2. Preparation of IPTG (100 mM)

1. In a 20 mL measuring cylinder, add 0.24 g of IPTG and raise the volume to 10 mL using molecular grade water.
2. Mix the content and make 1 mL aliquots in 1.5 mL microcentrifuge tubes. Store the content at −20 °C.

#### 5.2.3. Preparation of X-Gal (20 mg/mL)

1. In a 20 mL measuring cylinder add 0.2 g of X-Gal and raise the volume to 10 mL using DMSO.
2. Mix the content and make 1 mL aliquots in 1.5 mL microcentrifuge tubes. Store the content at −20 °C.

#### 5.2.4. Preparation of LB Agar Plates and LB Broth

1. Add the reagents mentioned in Table 28 and Table 29 in 200 mL conical flasks:
  For LB broth (100 mL)
  For LB agar (100 mL)
2. Aliquot the LB broth in 50 mL glass tubes. Cotton plug the LB agar flask and LB broth tubes and autoclave at 121 °C for 20 min.
3. LB broth tubes can be stored at room temperature or 4 °C for up to two months.
4. Prepare LB agar plates by transferring 20 mL LB agar into Petri plates inside biological safety cabinet.
5. LB agar plates can be stored at 4 °C for up to two months.

## Figures and Tables

**Table 1:** Experimental stages and time needed to complete every stage.

Experimental Stages	Time for Completion
1. Design, synthesis and verification of sgRNA	3 days
1.1. Design and ordering oligonucleotides	1 day
1.2. Synthesis and purification of sgDNA	3 h
1.3. Synthesis of sgRNA	18 h
1.4. Purification of sgRNA	1 h
1.5. In vitro verification of sgRNA	4 h
2. Injection of sgRNA-Cas9 mixture into embryos	1 day
2.1. Preparation of sgRNA-Cas9 mixture	15 min
2.2. Needle preparation	5 min
2.3. Collection of embryos	1 h
2.4. Arranging embryos in double-sided tape	15 min
2.5. Injection of sgRNA-Cas9 mixture	1 h
2.6. Incubation of injected embryos	10 min
3. Rearing of hatched individuals	4–5 weeks
3.1. Embryonic stage	3–4 days
3.2. Larval stage	3 weeks
3.3. Pupal stage	1–2 weeks
4. Genotyping of mutant individuals	3 days
4.1. Isolating DNA from mutant clones	1 day
4.2. Amplification of region of interest	3 h
4.3. T7 endonuclease assay on the region of interest	2 h
4.4. Cloning of the region of interest	1 day
4.5. Isolating plasmids from clones	1 h
4.6. Sequencing region of interest	5 h
4.7. Analyzing the sequencing data	1–2 h
● Synthesis of Cas9 mRNA (optional)	1 day

Note: The template DNA for synthesizing sgRNA (single guide RNA) is called sgDNA (single guide DNA).

**Table 2:** Primers for sgDNA synthesis.

Primer	Sequence
Forward primer for En1_guide1(En1_G1)	GAAATTAATACGACTCACTATAGGAGACCGTTGCAGTCCGAAC CGTTTTAGAGCTAGAAATAGC
Forward primer for En1_guide2(En2_G2)	GAAATTAATACGACTCACTATAGGGTTTGACAGAACGATGACA GGTTTTAGAGCTAGAAATAGC
Reverse primer for guide synthesis (CRISPR_R)	AAAAGCACCGACTCGGTGCCACTTTTTCAAGTTGATAACGGACT AGCCTTATTTTAACTTGCTATTTCTAGCTCTAAAAC

**Table 3:** Primers for amplifying DNA of interest (En1_E1_5′).

Primer	Sequence
En1_Exon1_with_5′UTR_Forward (En1_E1_5′_F)	GCGCATCGTGTTGTCAAAAA
En1_Exon1_with_5′UTR_Reverse (En1_E1_5′_R)	TAGATTGCTGTTCCCGCTTT
M13F	CGCCAGGGTTTTCCCAGTCACGAC
M13R	TCACACAGGAAACAGCTATGAC

**Table 4:** Reaction mixture for sgDNA synthesis.

Reagents	Volume (µL)
Q5 high fidelity DNA polymerase	1
Q5 polymerase buffer 10×	20
dNTP mix	2
Forward Primer (En1_G1)	3
Reverse Primer (CRISPR_R)	3
Molecular grade water	71

dNTP: Deoxynucleotides.

**Table 5:** Polymerase chain reaction (PCR) settings for sgDNA synthesis.

Temperature (°C)	Time (s)	Number of Cycles
98	30	1
98	$\begin{array}{l} 10\\ 30\\ 15 \end{array}\}$	30
57		
72		
72	120	1
4	∞	1

**Table 6:** Reaction mixture for sgRNA synthesis.

Reagents	Volume (µL)
T7 10× buffer	2
T7 RNA polymerase	2
ATP, GTP, UTP, CTP (10 mM)	8 (2 µL each)
sgDNA (500 ng/µL)	2
Ribolock	0.5
Molecular grade water	5.5

**Table 7:** Reaction mixture for Cas9-mRNA synthesis.

Reagents	Volume (µL)
Template DNA	2
2× NTP (provided in the mMESSAGE mMACHINE kit)	10
10× buffer (provided in the mMESSAGE mMACHINE kit)	2
T3 enzyme (provided in the mMESSAGE mMACHINE kit)	2
Ribolock	0.5
Molecular grade water	3.5

NTP: Nucleotide triphosphate

**Table 8:** Reaction mixture of Cas9-mRNA poly(A) tailing.

Reagents	Volume (µL)
10 mM ATP (provided in Poly(A) Tailing kit)	10
25 mM MnCl_2_ (provided in Poly(A) Tailing kit)	10
2× buffer (provided in Poly(A) Tailing kit)	20
E-PAP (provided in Poly(A) Tailing kit)	4
Molecular grade water	30

E-PAP: *Escherichia coli* Poly(A) Polymerase I

**Table 9:** Reaction mixture for amplification of DNA of interest.

Reagents	Volume (µL)
2× PCRBIO Taq Mix Red	12.5
Forward Primer (En1_E1_5′_F)	1
Reverse Primer (En1_E1_5′_R)	1
Template (gDNA/plasmid/cDNA)	1
Molecular grade water	9.5

**Table 10:** PCR condition for amplifying DNA of interest.

Temperature (°C)	Time (s)	Number of Cycles
95	60	1
95	$\begin{array}{l} 15\\ 15\\ 15 \end{array}\}$	40
Gradient (55–65)		
72		
4	∞	1

**Table 11:** Reaction mixture for sgRNA invitro verification.

Reagents	Volume (µL)
Cas9 buffer 10×	1
Cas9 Protein NLS (3300 ng/µL)	0.1
sgRNA (600 ng/µL)	0.5
Molecular grade water	8.4

**Table 12:** Reaction mixture for alternative sgRNA in vitro verification.

Reagents	Volume (µL)
NEB buffer 3.1	1
sgRNA (600 ng/µL)	0.5
Molecular grade water	8.4

**Table 13:** (a) Program settings for needle pulling (1st heat-pull cycle); (b) program settings for needle pulling (2nd heat-pull cycle); (c) program settings for needle pulling (3rd heat-pull cycle).

**a**	
**Conditions**	**Value**
Heat	625
Pull	10
Vel	10
Time	250
**b**	
**Conditions**	**Value**
Heat	625
Pull	10
Vel	10
Time	200
**c**	
**Conditions**	**Value**
Heat	625
Pull	10
Vel	10
Time	150

Note: Time value determines the duration of cooling (200 is equivalent to 100 milliseconds). Since the glass thickness reduces after every cycle, the time value is gradually decreased. The units of heat, pull and vel are mentioned in ref [62].

**Table 14:** Reaction mixture for embryo injections.

Reagents	Volume (µL)
Cas9 buffer 10×	1
Cas9 Protein NLS (3300 ng/µL)	1
sgRNA (600 ng/µL)	5
Molecular grade water	3

**Table 15:** Reaction mixture to amplify DNA of interest from mutant tissue DNA.

Reagents	Volume (µL)
2× PCRBIO Taq Mix Red	12.5
Forward Primer (En1_E1_5′_F)	1
Reverse Primer (En1_E1_5′_R)	1
Template (gDNA/plasmid/cDNA)	1
Molecular grade water	9.5

**Table 16:** Gradient PCR settings to amplify DNA of interest from mutant tissue DNA.

Temperature (°C)	Time (s)	Number of Cycles
95	60	1
95	$\begin{array}{l} 15\\ 15\\ 15 \end{array}\}$	40
Gradient (55–65)		
72		
4	∞	1

**Table 17:** Reaction mixture for T7 endonuclease verification.

Reagents	Volume (µL)
Amplified DNA (200 ng/µL)	1
10× NEB Buffer 2	2
Molecular grade water	17

**Table 18:** PCR settings for T7 endonuclease hybridization.

Temperature (°C)	Time (s)
95	300
95–85	5
85–25	600
8	∞

**Table 19:** Reaction mixture for ligating DNA of interest to plasmid.

Reagents	Volume (µL)
2× Rapid ligation buffer	5
pGEM-T vector	0.5
Amplified DNA (200 ng/µL)	0.5
T4 DNA ligase	1
Molecular grade water	3

**Table 20:** Reagents for the screening of positive bacterial colonies.

Reagents	Volume (µL)
IPTG	25
X-GAL	25
Ampicillin	25

**Table 21:** Reaction mixture to verify DNA inserted into plasmid.

Reagents	Volume (µL)
2× PCRBIO Taq Mix Red	12.5
M13F primer	1
M13R primer	1
Homogenized clone	1
Molecular grade water	9.5

**Table 22:** PCR settings to amplify inserted DNA along with the M13 region of the plasmid.

Temperature (°C)	Time (s)	Number of Cycles
95	60	1
95	$\begin{array}{l} 15\\ 15\\ 15 \end{array}\}$	30
57		
72		
4	∞	1

**Table 23:** Reaction mixture for sequencing forward strand of the cloned DNA.

Reagents	Volume (µL)
Plasmid	1
M13F primer	3
BigDye Terminator v3.1	0.5
5× BigDye buffer	2
Molecular Grade Buffer	3.5

**Table 24:** Reaction mixture for sequencing reverse strand of the cloned DNA.

Reagents	Volume (µL)
Plasmid	1
M13R primer	3
BigDye Terminator v3.1	0.5
5× BigDye buffer	2
Molecular Grade Buffer	3.5

**Table 25:** PCR settings for sequencing.

Temperature (°C)	Time (s)	Number of Cycles
96	60	1
96	$\begin{array}{l} 10\\ 5\\ 120 \end{array}\}$	30
54		
60		
4	∞	1

**Table 26:** Number of hatchlings in five experiments using 100 ng/µL and 300 ng/µL of En1_G2 sgRNA.

Experiment	Gene (Date/Month)	Eggs Injected	Hatchlings	% Hatching
1	En1 (4 August 2017) 300 ng/µL with Cas9 protein	280	16	5.71
2	En1 (22 October 2017) 100 ng/µL with Cas9 protein	187	18	9.63
3	En1 (23 September 2017) 300 ng/µL with Cas9 protein	272	35	12.86
4	En1 (15 February 2018) 300 ng/µL with Cas9 mRNA	164	14	8.54
5	En1 (1 March 2018) 300 ng/µL with Cas9 protein	352	168	47.72

Note: The number of hatched individuals during the early phase of experiments was low, most probably due to improper needle tip thickness, resulting in a large hole and embryo dehydration.

**Table 27:** Reagents for 50× TAE buffer preparation.

Reagents	Weight/Volume
Trizma base	242 g
Disodium EDTA	18.61 g
Glacial Acetic Acid	57.1 mL

**Table 28:** Reagents for 100 mL Luria–Bertani (LB) broth preparation.

Reagents	Weight/Volume
LB base	2.5 g
MilliQ water	100 mL

**Table 29:** Reagents for 100 mL LB agar preparation.

Reagents	Weight/Volume
LB agar	3.2 g
MilliQ water	100 ml

## Supplementary Materials

A supporting Videoarticle is available online at https://zenodo.org/record/1248482#.Wv1AASCbyUk.
